# Feasibility and effects of preventive home visits for at-risk older people: Design of a randomized controlled trial

**DOI:** 10.1186/1471-2318-9-54

**Published:** 2009-12-03

**Authors:** Malcolm P Cutchin, Susan Coppola, Vibeke Talley, Judie Svihula, Diane Catellier, Kendra Heatwole Shank

**Affiliations:** 1 Division of Occupational Science, Department of Allied Health Sciences, University of North Carolina, Chapel Hill, North Carolina, USA; 2 Orange County Department on Aging, Hillsborough, North Carolina, USA; 3 Institute on Aging, University of North Carolina, Chapel Hill, North Carolina, USA; 4 Department of Biostatistics, University of North Carolina, Chapel Hill, North Carolina, USA

## Abstract

**Background:**

The search for preventive methods to mitigate functional decline and unwanted relocation by older adults living in the community is important. Preventive home visit (PHV) models use infrequent but regular visits to older adults by trained practitioners with the goal of maintaining function and quality of life. Evidence about PHV efficacy is mixed but generally supportive. Yet interventions have rarely combined a comprehensive (biopsychosocial) occupational therapy intervention protocol with a home visit to older adults. There is a particular need in the USA to create and examine such a protocol.

**Methods/Design:**

The study is a single-blind randomized controlled pilot trial designed to assess the feasibility, and to obtain preliminary efficacy estimates, of an intervention consisting of preventive home visits to community-dwelling older adults. An occupational therapy-based preventive home visit (PHV) intervention was developed and is being implemented and evaluated using a repeated measures design. We recruited a sample of 110 from a population of older adults (75+) who were screened and found to be at-risk for functional decline. Participants are currently living in the community (not in assisted living or a skilled nursing facility) in one of three central North Carolina counties. After consent, participants were randomly assigned into experimental and comparison groups. The experimental group receives the intervention 4 times over a 12 month follow-up period while the comparison group receives a minimal intervention of mailed printed materials. Pre- and post-intervention measures are being gathered by questionnaires administered face-to-face by a treatment-blinded research associate. Key outcome measures include functional ability, participation, life satisfaction, self-rated health, and depression. Additional information is collected from participants in the experimental group during the intervention to assess the feasibility of the intervention and potential modifiers. Fidelity is being addressed and measured across several domains.

**Discussion:**

Feasibility indications to date are positive. Although the protocol has some limitations, we expect to learn enough about the intervention, delivery and effects to support a larger trial with a more stringent design and enhanced statistical power.

**Trial Registration:**

ClinicalTrials.gov ID NCT00985283

## Background

The current state of affairs in population aging and gerontology in the USA and abroad set the stage for this study protocol. While an extraordinarily large cohort of the population is moving into the latter phases of life, the resources to fund both acute and long term care for that cohort are in question. Gerontological research suggests that this cohort will want to remain at home and their well-being can best be maintained by doing so [1,2]. At the same time, an emerging understanding of older adult well-being suggests that domains of activity, participation, and overall engagement with life are essential. In light of these circumstances, there is a distinct need to develop a solution that will enhance the well-being of older adults in these domains and allow them to stay at home longer as well as remain functional, relatively healthy and satisfied. The project described here intends to begin the process of addressing these needs by testing an intervention termed *preventive home visits *(PHVs).

The USA and other countries are confronted with the responsibility of not only taking care of the growing population of older adults who become sick or frail but also helping those who are not yet in need of care to maintain their independence and well-being. Effective prevention could produce health care cost savings as well as improve the lives of older adults and their families. A particular goal of prevention in the older population, therefore, is providing older adults the ability to remain in their own home and community instead of entering the formal care system. As aging progresses, the probability of frailty or sickness, and thus separation from home, increases. Such separation can be traumatic for older adults. On the other hand, the ability to remain at home can help an older adult maintain mental and physical health through engagement in daily activities—such as housekeeping or social interactions—if adaptations to the aging process can be learned and used.

The argument here is two-fold. First, the gerontological evidence is strongly suggestive that a positive outlook on life (e.g., affect, happiness, optimism) can enhance mental and physical well-being [3]. Second, the ability to engage mentally, socially, and physically with the world from a community setting can positively affect both sides of the outlook and health equation [4-8]. Moreover, extending independence and participation for older adults can have important positive consequences for others. Informal caregivers (e.g., children of older adults) carry a large burden and suffer their own negative health consequences when older adults become frail and ill [9]. A program that could affect older adults and their family and care networks would extend beyond the issue of individual betterment to community improvement. To be able to sustain or improve participation, function, and social interaction within the context of aging in one's own home and community setting is therefore an admirable and compelling goal.

Based on systematic reviews and meta-analyses, evidence about effectiveness is mixed but supportive enough to suggest continued research. The first comprehensive review of programs similar to preventive home visits was conducted by Stuck and Siu [10] who concluded that comprehensive geriatric assessment (CGA) programs were associated with statistically significant higher odds of living at home at follow-up. In a similar review of 21 randomized controlled trials of "health assessments" Byles [11] concluded that such interventions were associated with improved health outcomes for older adults but that specific mechanisms for those associations were difficult to identify. Van Haastregt and colleagues [12] focused their systematic review more specifically on 15 studies of preventive home visits. Although the authors noted substantial differences among the interventions, samples, and outcome measures and that visits were more often than not tailored to the needs of participants in the studies reviewed, they concluded that there was no clear evidence for the efficacy of such visits. In another systematic review soon after, Elkan et al. [13] criticized van Haastregt and colleagues for not pooling the results of the studies they reviewed. The results of their 15 study meta-analysis focused on preventive home visits by trained nurses and suggested that they are associated with a decrease in mortality and a reduction in admissions to long term care institutions. Assessing multidimensional preventive home visit programs since 2000, Huss et al. [14] concluded that such programs have positive effects on mortality rates, functional decline, and nursing home admissions, especially in younger old groups (77 or younger). A large study in Denmark using pre-existing home visit programs in 34 municipalities with over 4000 older people established that an educational intervention used with the home visits (to improve skills, interdisciplinary collaboration and reduce ageism among providers) had the positive outcomes of improved functional ability and cost neutrality [15,16].

The one systematic review of 17 occupational therapy interventions for community dwelling older people [17] argues that such interventions result in various positive outcomes. The types of interventions reviewed included comprehensive OT interventions (outside the home), training of skills combined with instruction on assistive devices, instruction only regarding assistive devices, and counseling of primary care providers. Most outcome measures used in the reviewed studies focused on activities of daily living or functional independence measures, and only a couple used measures of well-being (e.g., life satisfaction) or general health outcomes (e.g., the MOS 36-Item Short-Form Health Survey SF-36). The one comprehensive OT intervention study that was of high quality and with positive results [18,19] used outcome measures for participation, function, life satisfaction, and health. More recently, Gitlin and colleagues [20] have suggested the feasibility of in-home OT-based prevention for older people with dementia and their caregivers. Our study builds on this evidence base for interventions outside the home and Gitlin and colleagues' suggestion of the feasibility of an in-home, OT-based preventive intervention.

### Aims and hypotheses

**Figure 1:**
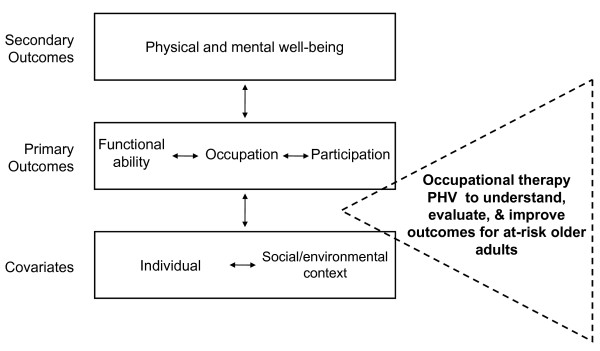
**Conceptual framework for preventive home visits study**.

We developed our approach using the research literature as well as principles of Danish preventive home visits [21]. We also used a conceptual framework synthesized from (a) the World Health Organization's (WHO) International Classification of Functioning, Disability, and Health, or ICF as it is widely known [22] and (b) the American Occupational Therapy Association's (AOTA) practice framework [23] (see Figure 1). Common in those frameworks is the perspective of *positive *function, aging, and participation. The primary aims were to (a) determine if the PHV intervention used in the proposed project is feasible in the USA context, (b) estimate the effect of a PHV intervention on functional ability, (c) ascertain if the PHV intervention causes improved psychosocial outcomes that relate to well-being, and (d) estimate the effect of the intervention on health outcomes. Accordingly, we used hypotheses such as the following examples to structure the measurement and analysis of outcomes: (*H*_1_) the experimental group will exhibit greater (more positive or less negative) functional ability across the study period than the comparison group, (*H*_2_) the experimental group will show a significantly better slope for life satisfaction across the study period than the comparison group, and (*H*_3_) the experimental group will have significantly lower rates of referral to rehabilitation, skilled nursing or assisted living facilities than the comparison group.

## Methods/Design

The study goals are to assess the feasibility, and to obtain preliminary efficacy estimates, of an occupational therapy-based preventive home visit (PHV) intervention for community-dwelling older adults. To achieve these goals, a single-blind randomized controlled pilot trial using a repeated measures design is employed. Community-living (not in assisted living or a skilled nursing facility) older adults (75+) in one of three central North Carolina counties found to be at-risk for functional decline were eligible for the study. Participants who consented were randomly assigned to an experimental or a comparison group. Over a 12-month follow-up period, the experimental group receives 4 preventive home visits while the comparison group receives a minimal intervention of mailed printed materials. Pre- and post-intervention questionnaire measures are administered face-to-face by a treatment-blinded research associate. Additional information is collected from participants in the intervention group during the intervention to assess the feasibility of the intervention and potential modifiers. The protocol was approved by the Public Health/Nursing Institutional Review board of the University of North Carolina at Chapel Hill. Written informed consent is used with all participants.

### Recruitment and randomization

**Figure 2:**
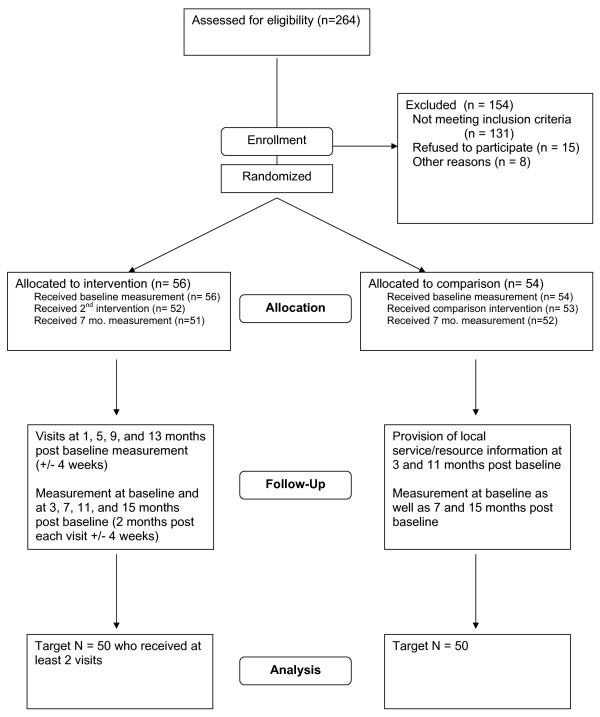
**Participant flow chart**.

We recruited the sample through several concurrent methods: senior center and clinic flyers, email to community-based lists, community presentations, random phone calls to those on local senior center rosters, and announcements via local aging service providers. For those who consented to continue through the recruitment process, we used the Vulnerable Elders Survey (VES) [24] to determine at-risk participants for inclusion in the study. To ensure that treatment groups were balanced on important potential confounders, we used permuted-block randomization within strata defined by VES scores (3-4 or 5+), ethnicity (non-Hispanic white or minority), and residential status (living alone or not) (see Table 1). The recruitment and allocation is further illustrated in Figure 2.

**Table 1:** Measurement process.

CONSTRUCTS	OPERATIONALIZATION	**TIME COLLECTED**^**#**^									
		**S**	**O**_**1**_	**I**_**1**_	**O**_2_^*^	**I**_**2**_	**O**_**3**_	**I**_**3**_	**O**_4_^*^	**I**_**4**_	**O**_**5**_

**Screening for Inclusion**											

Vulnerability	Vulnerable Elders Survey (VES)	X									

**Blocking/Randomizing**											

VES score	3-4, 5+	X									
Ethnicity	White, other		X								
Living arrangement	with someone, alone		X								

**Outcomes**											

*Primary Outcomes*											
Functional ability	Late Life Function & Disability Instrument		X		X		X		X		X
Participation	Late Life Function & Disability Instrument		X		X		X		X		X
Occupation	self-assessed performance and frequency			X		X		X		X	

*Secondary Outcomes*											
Life satisfaction	Satisfaction with Life Scale		X		X		X		X		X
General health	SF-12 v.2		X		X		X		X		X
Depression symptoms	CESD-10		X		X		X		X		X

*Tertiary Outcomes*											
Acute care utilization	frequency, duration		X		X		X		X		X
Rehab or longer term care	frequency, duration		X		X		X		X		X

**Covariates^**											

*Demographics*											
Age	years		X								
Gender	male, female		X								
Ethnicity	White, African Amer., Hispanic, Asian, oth		X								
Education level	years completed		X								

*Environment of the Home*											
Type of dwelling	single family, duplex, apt., mobile, other		X								
Location of dwelling	city, suburban, rural		X								
Problems with steps	none, minor, moderate, major			X							
Problems with indoor lighting	none, minor, moderate, major			X							

*Social/Cultural Context*											
Living companion	alone, spouse, child, other etc.		X								
Knows neighbors	well, somewhat, little, no contact		X								
Interaction with family	days per week			X							
Importance of spirituality/religion	very, somewhat, not			X							

*Health & Function*											
Self-rated energy level	low (1) - high (5)			X		X		X		X	
Pain	low (1) - high (5)			X		X		X		X	
Fall frequency	number of falls in past 4 months			X		X		X		X	
Cognitive function	6-Item Cognitive Impairment Test		X		X		X		X		X

*Community Mobility*											
Accessing community	frequency; purpose			X							
Driving status	yes, with limits, ceased			X							
Public transportation	availability; use; type			X							

*Adherence to recommendations*	to all, to roughly half, to none					X		X		X	

**Fidelity Dimensions**		**P**									

Created manual		X									
Protocol manual training for OTs	checklist for competence	X									
Clinical/case meetings	maintaining reliability of intervention				X		X		X		
Intervention Delivery Checklist	administers protocol as trained? (Yes, No)			X		X		X		X	
Participant exit interview	participant perceptions of intervention and own behaviors vis-à-vis recommendations										X


^# ^P = pre-study, S = screen for inclusion, O = measurement occasion by researcher, I = intervention delivery and data collection by OT

* Observations conducted by telephone

^ Constructs listed under these categories are examples and not inclusive of all being measured

### Procedure

#### Experimental group

The intervention is a structured protocol that leaves room for flexibility based on the older adult and his or her situation, preferences, and needs. Therefore, it is uniform in terms of the domains that are evaluated by the visiting occupational therapist (OT), but the recommendations are individualized (tailored) for each participant. Core domains covered in each visit include (a) physical environment of home and surroundings, (b) social/cultural context, (c) performance and participation in occupations, (d) health and functional concerns affecting participation, and (e) community mobility issues affecting participation. At each PHV, the occupational therapist makes sure that all domains are covered before leaving the participant's home, but the structure of the visit may vary because we believe it is essential for the therapist to listen actively to the participant and let the participant lead the direction of the conversation and activity to areas important to her or him. The visit is a collaborative effort and the participant is encouraged to be as active as possible in identifying both health promoting activities and problem areas. The OT and the participant work together to develop solutions. Before leaving, the OT reviews the list of issues and health promoting activities with the participant and describes in general terms which areas the recommendations will address. After the visit, the OT generates up to 5 detailed key recommendations and shares them with the subject via mail. The second through fourth visits are additional assessments with more focus on how the recommendations have worked, changes in the participant's situation, and what recommendations are needed at that point.

#### Comparison group

In order to be able to attribute treatment effects to the tailored and OT-delivered PHV intervention, we use "attention" control, i.e., a generic, minimal intervention providing nonspecific attention and support by non-occupational therapy personnel. This minimal intervention consists of a mailed information packet about local services for older adults as well as information on fall prevention from the Centers for Disease Control and Prevention. The intervention was devised because it is a low cost program that any local Department on Aging or senior center should be able to offer to community dwelling older adults.

### Measures

Table 1 presents study constructs, their operationalization as measures, and the pattern of their collection during the study. In that table, we indicate screening measures, measurement observations conducted by a blinded research associate, and measures collected by OTs during their intervention visits.

#### Primary outcomes

We are using the function component of the Late Life Function and Disability Instrument (LLFDI) to measure the functional ability constructs [25]. This 32 item scale measures physical function in a comprehensive manner, and captures 3 dimensions of function: advanced lower extremity functioning, basic lower extremity functioning, and upper extremity functioning. The dimensions provide measures of functional ability that we expect to be associated both with performance of occupations and participation and which may be affected by the intervention. Cronbach's alphas for the 3 dimensions range from .86-.96, and test-retest ICCs above .90 were reported by the authors.

The study also employs the disability component of the LLFDI to measure participation. Although we wish the authors had used the more positive language of the ICF—participation, instead of disability—the instrument measures participation. This 16 item scale contains two dimensions. The Social Role dimension (this equates with our definition of participation) items begin with words such as "visit, ""take part in, ""invite,""go out with, " and "keep in touch" that strongly emphasize participation in social affairs and interactions. Personal Role (to "take care of" own health, home, meals, etc.) are similar to activities of daily living. All items are based on a measure of the frequency and limitation of participation ("performance" in the authors' parlance). Jette et al. [26] report reliability evidence and Rasch models for the participation dimensions that support a unidimensional (one-factor; i.e., combining the two sets of factors for one summary score) interpretation of participation. We will take the one-factor approach to create two participation variables, one each for frequency and limitation. As reported by Jette and colleagues, the Cronbach alpha for the one-factor solution of participation frequency was .82, and the internal consistency for participation limitation was .92. Test-retest reliabilities were acceptable and discriminant validity evidence was encouraging.

Occupational performance data are collected regarding type of occupations desired by the older person as well as frequency of performance (ordinal scale: 4 = As often as I would like, 3 = almost as often as I would like, 2 = less than I like, 1 = never) and quality of performance (2 = no problem in performance, 1 = problem in performance) are collected during the first OT visit. Frequency and quality measures are coded by the OT using information provided by an interview at subsequent visits. Occupation is measured in the experimental group only.

#### Secondary and tertiary outcomes

Life satisfaction is a global construct within the realm of subjective well-being, and it can be understood to encompass both emotional and judgmental evaluation of life [27]. Most measures tend toward the emotional component that relates to happiness and affect. The Satisfaction with Life Scale (SWLS) [28] is one of the few measures of the judgmental dimension of well-being and is designed for adults of all ages [29]. This brief, 5 item instrument uses a 7 point Likert-type response scale yielding possible scores from 5-35. The instrument yields a uni-dimensional measure with high internal consistency (Cronbach's alpha ≥ .80), good test-retest reliability, and studies report various forms of good validity evidence [30].

The concept of health is complex, and although we take the position that physical and mental health are intimately related, the idea to break the measurement of health into physical and mental components is worthwhile because causes and effects may show first in one component or the other. A well regarded and widely used instrument for measuring overall health and its two primary components is the SF-36 Health Survey. The second version of that instrument [31] is now available as a revised version of a brief form, the SF-12 v.2. The briefer SF-12 and its two component scores for overall physical and mental health have shown psychometric properties (internal consistency, test-retest reliability, validity) similar to those of the longer SF-36 [32]. As with the SF-36, the scoring algorithm uses item weighting and component scores are scaled to match the SF-36 scores. The SF-12 does not have the sensitivity to more specific dimensions captured in the longer version, but its utility to provide quality measures of overall mental and physical health status in a brief format (twelve items) makes it ideal for research with older adults. We will use the general health score as well as the physical and mental component scores as health outcome variables.

In addition to the mental health measures on the SF-12 we found it important to screen for depression in the participants. Depression is a significant issue in older adults affecting initiative, participation and activity. Chachamovich et al. [33] found that even minor depression is associated with a significant decrease in all aspects of quality of life. The Center for Epidemiologic Studies Short Depression Scale (CES-D 10) is frequently used with well older adults as a screening tool for depression and has shown very good reliability, validity, specificity and sensitivity [34,35]. It is brief with scores ranging from 0-30. A score of 10 or greater is considered depressed. This measure will be used as a secondary outcome variable.

Tertiary outcome measures include information about acute health care utilization as well as rehabilitation and longer term care setting utilization. At each measurement occasion we ask all participants, regardless of group assignment, to report on the frequency and/or duration of admissions to a hospital, an emergency room, a rehabilitation facility, a skilled nursing facility, as well as urgent physicians' visits.

#### Covariates

The PHV instrument, used by the OTs during in-home visits, includes a variety of personal and situational variables into the study. Individual variables collected with that instrument include: age, gender, ethnicity/race, and education. Contextual measures include home environment items such as type of dwelling, location, and environmental obstacles such as steps or lighting. Social and cultural context variables such as living companion, how well one knows neighbors, frequency of interaction with family, and the importance of spirituality, are captured via self report with this instrument by the OTs. The visiting therapists also ask for self reports of energy level, pain, and fall frequency, and they inquire about and record community mobility measures. Although Table 1 does not reflect it, changes in these domains are noted in the PHV instrument during visits 2-4, and they can be coded as negative or positive and included as additional covariates if needed.

In addition to those measures collected by visiting therapists, the data collection research associate uses the 6-item Cognitive Impairment Test (6CIT), also known as the Short Orientation-Memory-Concentration Test (SOMCT) to measure cognitive function. The 6CIT provides a score ranging from 0-28 [36]. This test has the advantage of being very brief, but it also is highly correlated (r = .92) with the longer Blessed Test, and it was validated with neuropathology results. The 6CIT instrument is highly correlated with the commonly used Mini Mental Status Exam (r= .91), but it shows much higher sensitivity to mild dementia, 78% vs. 51% [37].

#### Feasibility and fidelity

Feasibility is being measured by the rate of recruitment, representativeness of the study sample, and retention rates. Fidelity is being measured in several dimensions suggested by Frank et al. [38]. In order to address fidelity to provider training, we created a manual and then used it to train two additional OTs in a brief course. That has been followed by a series of clinical meetings of the OTs in which cases and intervention strategies are discussed and refined. To address fidelity to treatment delivery, we have used independent, trained observers to rate OT adherence to the major dimensions of the intervention protocol. Fidelity to receipt of treatment and treatment enactment is measured during participant exit interviews.

### Analysis plan

Descriptive statistics (means, proportions, and associated confidence intervals) will be used to summarize measures of feasibility and fidelity. In order to establish if a larger-scale trial is warranted and to help estimate the sample size needed for such a trial, we will estimate the efficacy of the PHV intervention. Those estimates will either strengthen the scientific rationale for proceeding with a full-scale trial, or cause us to revise current projections for treatment effects. It is important to note that as a result of the small sample size, and the short length of the intervention, the estimates of the intervention effects will be imprecise. Efficacy analyses will include treatment group comparisons at 7 and 15 months with respect to measures of life satisfaction and general health status. General linear models (GLM) will be used to estimate the difference in the means for these continuous outcomes (LLFDI function and disability summary scores, SF-12 physical and mental health summary scores). In addition to comparisons at discrete time points, repeated measures analysis of variance will be used to compare the pattern of change over the entire period of follow-up. Differences between intervention and comparison participants on health care utilization will be tested by both chi-square tests and logistic regression analysis. Secondary analyses will consider models that adjust for potential confounding due to imbalances in group size. Finally, there is likely to be some variation in the dose of the intervention received across all participants in the study. To investigate whether efficacy is affected by dose, we will compare outcomes across groups defined by the number of PHV visits received using the models specified above.

Measures for cost effectiveness evaluation will come from travel and time data logged by the visiting OTs. Administrative expenses (scheduling visits, calls, letters, etc.) are being closely tracked. These data will be combined with US salary norms for occupational therapists to estimate costs per participant intervention. In addition, these intervention cost data will be compared to medical care costs estimated by care utilization rates (tracked with the associated instrument) multiplied by standardized estimates of cost by care type. This will allow us to estimate cost differentials and savings produced by the intervention.

The conceptual model which forms the basis of the proposed intervention involves many complex inter-relationships between the primary, secondary, and tertiary outcomes. We acknowledge that the limited sample size of this trial restricts our ability to test complex mediational effects hypothesized by the PHV model framework. Nevertheless, we will explore simple relationships involving no more than four variables using structural equation models (SEM).

Efficacy of the PHV intervention will be evaluated with respect to a variety of outcomes, but the sample size for the full-scale trial will need to be computed for a single primary outcome. The choice of an outcome measure to use as primary will be based in part on efficacy results of this pilot study.

#### Study progress

The target sample size of 120 participants was selected to give the study 80% power to detect a difference of 0.5 standard deviation units on measures of functional ability and life satisfaction. Using the terminology of Cohen [39] this effect size can be considered "moderate." This corresponds, for example, to absolute differences of 4.0 and 7.4 units on the LLFDI frequency and limitation summary scores, and 5.7 units on the physical and mental health components of the SF-12. A total of 110 participants (92% of target) were enrolled (see Figure 2) over a 9 month period. The study sample is fairly representative with respect to gender (70% female), but is more highly educated (94% high-school education or higher) and less racially/ethnically diverse (92% white) than the general population of older adults in the counties targeted in this study. As of August 2009, 88% of participants have completed the 7-month follow-up visit, and 24% have completed the final closeout visit. We plan to complete all follow-up measurements by May 2010. Dissemination of results is planned for late 2010.

## Discussion

The potential limitations of the study are several. Although logically sound based on the literature and our experience with older adults, our protocol has not been proven feasible or effective, and this project will provide a good initial test. One potential challenge to analysis is that the intervention is delivered as a bundle, and that makes it difficult to pinpoint the cause of any effects. We believe that the embracing of complexity in older adults' lives is worth this limitation, however, and we are collecting detailed data through the PHV instrument to help us parse out effects. An additional limitation of our intervention is that it does not address directly the physical health limitations of older adults. Because the intervention is not a medical approach, we are not able to, nor are we attempting to, directly (medically) assist older adults with physical ailments. The visiting OT does, however, refer an older adult to medical care (or contact the appropriate agent about such need) if an older adult is experiencing acute health or safety problems or is clearly at-risk for near-term adverse health consequences due to a mental, physical or social problem. Because of budget constraints and the need to increase statistical power, a study design providing more interpersonal contact with the experimental group is potentially confounding. This was a compromise we found acceptable at this stage of the protocol's development and assessment. Future studies will balance group contact as well as group covariate measurement to allow for more stringent tests of effects.

## Competing interests

The authors declare that they have no competing interests.

## Authors' contributions

MPC led the research design and implementation of the study and was the primary author of the manuscript. SC participated in study design and co-developed the intervention. VT participated in study design, co-designed the intervention, and oversaw intervention implementation. JS participated in study design as well as data collection and management. DC participated in study design and analysis design. KHS developed the table. All authors contributed to the writing and review of the manuscript and approved the final manuscript.

## Pre-publication history

The pre-publication history for this paper can be accessed here:

http://www.biomedcentral.com/1471-2318/9/54/prepub
